# Promoting dignity as a core concept of healthcare: An opinion piece from an occupational therapy perspective

**DOI:** 10.3389/fresc.2026.1818009

**Published:** 2026-05-08

**Authors:** Amani Abdullatif Alnamnakani

**Affiliations:** Occupational Therapy Department, Batterjee Medical College, Jeddah, Saudi Arabia

**Keywords:** dignity, healthcare, occupational therapy, rehabilitation, wellbeing

## Introduction

Who does not want to be treated with dignity? When asking Google this question, the answer that was returned was “an artificial intelligence (AI) overview is not available for this search.” Certainly, everyone wants their dignity to be maintained, respected, and enhanced, irrespective of their status or condition (1–3). Dignity is often characterized as a complex concept (4). It concerns the identity that an individual possesses for some reason within the social community. It is how individuals perceive their worth within their community and how they perceive the community to be responding to this expression of identity (3, 5).

Dignity, in other words, can be described from the following two perspectives: intrinsic and extrinsic dignity (4). Intrinsic dignity refers to the inherent personal sense of self-worth that is given to all human beings when they are born by virtue of being human. In contrast, extrinsic dignity refers to the acquired sense of self-worth that can be honored or violated through the individual's interaction with others in a social context (4, 6).

The experience of dignity is significantly meaningful in people's lives, as it is rooted in values such as freedom, duty, responsibility, and serving others (2, 7). However, as human beings have a special existential dependency on being seen and taken care of by others, vulnerability is always a part of their life (7). As such, once a person becomes ill, their dignity is at stake (8). Violation of dignity, or indignity, refers to a situation in which someone's sense of self-worth, respect, or honor has fallen short of expectations and is compromised or diminished (5, 9). This can happen in several ways, such as through mistreatment, humiliation, demoralization, negligence, or being forced to compromise one's worth and values (4, 5, 10).

## Dignity in the context of healthcare and occupational therapy: facts and examples

The term “dignity” is increasingly becoming a part of contemporary discussions in everyday healthcare. Phrases such as “dignity in care,” “living with dignity,” and “death with dignity” have become commonplace (11–13). According to Ekpenyong et al. (1), independence is a safeguard of patient dignity; the more dependent a patient is on others, the more vulnerable they are to a loss of dignity.

Chochinov et al. (14) found that patients who report more difficulties with self-care activities are more likely to feel depressed, regard themselves as having become a burden to others, and lose their will to live. This is because the body can often be understood as either a symbol of dignity or a source of violation when bodily changes make it impossible to perform in accordance with the culture's dignity norms (7). Solomon et al. (15), however, indicated that clinically meaningful enhancements in one’s sense of dignity can be achieved with appropriate rehabilitation.

Rehabilitation is a highly person-centered healthcare approach, with a tailored set of interventions focusing on restoring a person's physical, mental, or cognitive abilities so they can lead a fulfilling life following a chronic illness or injury (16). It usually includes interventions provided by rehabilitation professionals such as physiotherapists, occupational therapists, speech and language therapists, psychologists, orthotic and prosthetic technicians, and physical and rehabilitation medicine doctors (16). Rehabilitation treatment often takes place through direct contact between the therapist and patient, such as in nursing and OT interventions.

Promoting dignity in nursing has various significant implications in an interdisciplinary rehabilitation context. This is because nursing staff provide the most frequent and close contact with patients requiring assistance with activities of daily living.

This means that dignity is not only shaped through formal therapeutic interventions but also through routine interactions such as respect for privacy, communication, and sensitivity during personal care. Therefore, it is essential that nursing staff are adequately supported through education and training so that they can understand and enact dignity-promoting practices in routine care (17). Without adequate education, training, and organizational support, there is a risk that dignity may be unevenly maintained, particularly in contexts of high workloads and task-focused care (18).

According to the World Federation of Occupational Therapists (WFOT), “Occupational therapy promotes health and wellbeing by supporting participation in meaningful occupations that people want, need, or are expected to do” [SIC] (19). This updated definition emphasizes the importance of participation in daily activities that are relevant to an individual's life, focusing on the *why* of occupation rather than just the *what* or *how* (19). Therefore, occupational therapists aim to personalize intervention plans to help patients perform their daily activities according to their needs. However, basic life activities such as eating, dressing, toileting, and showering require either hands-on techniques to practice fine motor skills or a therapist physically manipulating or directing the patient's body to support therapy goals.

What, then, are the implications of this direct contact on the virtue of dignity? One important implication is that healthcare professionals should show respect to their patients in ways that acknowledge the inherent worth of the individual in action, not just as a feeling. According to Jones (3), there are some ways in which respect can be shown, for example, by sitting in a chair or kneeling next to a patient rather than standing over them or by apologizing for any lack of courtesy one has shown in the past. Jones (3) contends that if respect is not shown in these ways, then all that the patient has to bear from ill health and healthcare professionals becomes a symbol of a lack of respect, a type of institutional humiliation.

The Dignity Project commenced in 2016 (20), which aimed to collect stories from citizens with disabilities to better inform clinical practice in rehabilitation, a field in which occupational therapists are often engaged. It revealed that citizens with acquired impairments, which develop during a person's lifetime, often experience breaches of dignity, and humiliation, and suffer inadvertent harm within systems of care (20, 21).

Another example was revealed during my doctoral research when I explored Zara's (pseudonym) experience within the healthcare system in the UK (33). Zara is a retired nurse who uses crutches and wears a niqab. She contracted human immunodeficiency virus (HIV) from her husband, and she was also diagnosed with a number of mental and physical health conditions. Zara described how she was frequently stared at by men when sitting in the waiting room at the HIV clinic because she is a woman, a Muslim, a niqab wearer, and an HIV-positive patient. Thus, Zara recounted a situation in which a patient's dignity was overridden in favor of institutional efficiency.

As a result, Zara described missing her appointments and not taking one pill, although she was taking 22 other pills a day, as a way to reduce her feeling of distress, despite acknowledging that this one pill *“*would save” her life (33, 2022, p. 120). Had the HIV clinic service providers engaged Zara in a conversation, they would have discovered why she had missed her appointments. Letting Zara wait in another area would be a simple solution to respect her intersectional identities and contribute to saving her life. This scenario, while specific, reflects a broader issue in healthcare: dignity is often compromised not merely through malevolence but through systemic regulations.

Another example that healthcare providers often encounter in their everyday practice is a patient who refuses treatment. Treatment refusal rates range from 5% to 25% in many studies (22–24). Despite a few studies (22–24) addressing this dilemma, the focus has been primarily directed at the patients, not the underlying issues in the system of care. For example, common reasons for refusal include fear, misinformation, financial concerns, physician advice, family burden, lack of capacity, and personal values and beliefs (25–28).

What is at stake here is that we often forget to consider that patients may refuse treatment if they feel a lack of dignity. In other words, if a patient refuses treatment, the refusal is tied to their autonomy and right to make informed decisions about their healthcare (28). However, Pirotte and Benson (28) contend that refusal of care does not indicate the end of healthcare providers’ responsibilities. They still need to advocate for the refusal decision and explain why those patients have refused care. As such, a special service should be allocated within each healthcare facility to address refusal concerns, similar to case management teams and early supported discharge services. The goal of this service would not be to change patients’ minds or pressure them into accepting care, but rather to ensure that all patients’ concerns, including dignified care, are addressed.

Moreover, per the World Health Organization (WHO), the International Classification of Functioning, Disability, and Health (ICF), the United Nations (UN) Convention on the Rights of Persons with Disabilities, and the World Report on Disability, rehabilitation services cannot remain limited to compensating for functional limitations and improving activities of daily living (29–31). Rather, they should increase the quality of life and dignity of consumers. Preserving patients’ dignity is imperative not only for their overall wellbeing but also for advancing the United Nations Sustainable Development Goals (SDGs), particularly SDG 3 (good health and wellbeing), SDG 5 (equality), and SDG 10 (reduced inequalities), which collectively emphasize inclusion, health equity, and social justice.

## Conclusion

All individuals have inherent worth and deserve to be treated with dignity. Dignity is central to people's lives, as it is rooted in values such as freedom, duty, and serving others. Illness can threaten a person's dignity, since it exposes their vulnerability. As such, direct therapist–patient contact requires showing respect in action through courtesy, empathy, unconditional positive regard, and specific physical postures to preserve dignity. Lack of respect or rigid systems can cause breaches of dignity and institutional humiliation, leading to a refusal of care. Establishing a dedicated service to handle treatment refusal can help uphold patient dignity and ensure all patients’ concerns are respectfully addressed. When patients feel their inherent worth is recognized, they experience less anxiety and are more likely to adhere to treatment plans, leading to better health and well-being outcomes. In the words of Robin Williams, playing Dr. “Patch” Adams in the film, *Patch Adams*, “You treat a disease: you win, you lose. You treat a person, I guarantee you, you'll win, no matter what the outcome” (32). This perfectly emphasizes the need to reposition dignity not as an abstract value, but as a core clinical imperative—one that occupational therapists are uniquely positioned to advocate and empower their patients for.
